# Development of a mathematical model for predicting electrically elicited quadriceps femoris muscle forces during isovelocity knee joint motion

**DOI:** 10.1186/1743-0003-5-33

**Published:** 2008-12-10

**Authors:** Ramu Perumal, Anthony S Wexler, Stuart A Binder-Macleod

**Affiliations:** 1 Department of Physical Therapy, University of Delaware, Newark, DE, USA; 2 Department of Mechanical and Aeronautical Engineering, Civil and Environmental Engineering, and Land, Air, and Water Resources, University of California, Davis, CA, USA

## Abstract

**Background:**

Direct electrical activation of skeletal muscles of patients with upper motor neuron lesions can restore functional movements, such as standing or walking. Because responses to electrical stimulation are highly nonlinear and time varying, accurate control of muscles to produce functional movements is very difficult. Accurate and predictive mathematical models can facilitate the design of stimulation patterns and control strategies that will produce the desired force and motion. In the present study, we build upon our previous isometric model to capture the effects of constant angular velocity on the forces produced during electrically elicited concentric contractions of healthy human quadriceps femoris muscle. Modelling the isovelocity condition is important because it will enable us to understand how our model behaves under the relatively simple condition of constant velocity and will enable us to better understand the interactions of muscle length, limb velocity, and stimulation pattern on the force produced by the muscle.

**Methods:**

An additional term was introduced into our previous isometric model to predict the force responses during constant velocity limb motion. Ten healthy subjects were recruited for the study. Using a KinCom dynamometer, isometric and isovelocity force data were collected from the human quadriceps femoris muscle in response to a wide range of stimulation frequencies and patterns. % error, linear regression trend lines, and paired t-tests were used to test how well the model predicted the experimental forces. In addition, sensitivity analysis was performed using Fourier Amplitude Sensitivity Test to obtain a measure of the sensitivity of our model's output to changes in model parameters.

**Results:**

Percentage RMS errors between modelled and experimental forces determined for each subject at each stimulation pattern and velocity showed that the errors were in general less than 20%. The coefficients of determination between the measured and predicted forces show that the model accounted for ~86% and ~85% of the variances in the measured force-time integrals and peak forces, respectively.

**Conclusion:**

The range of predictive abilities of the isovelocity model in response to changes in muscle length, velocity, and stimulation frequency for each individual make it ideal for dynamic applications like FES cycling.

## Introduction

**Figure 1:**
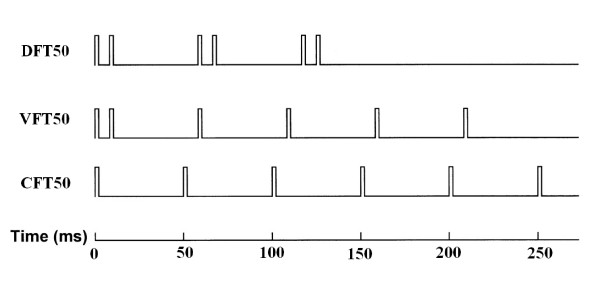
Schematic representation of the three stimulation patterns used. Bottom train (CFT50) is a constant-frequency train with all interpulse intervals equal to 50 ms; middle train (VFT50) is a variable-frequency train with an initial doublet of 5 ms and remaining pulses equally spaced by 50 ms; and top train (DFT50) is a doublet-frequency train with 5-ms doublets separated by interdoublet interval of 50 ms. Each train's name is based on the duration of the longest interpulse interval within that train. Each train has a maximum of 50 pulses (not shown in figure) and a pulse width of 600 *μ*s.

Functional Electrical Stimulation (FES) is the coordinated electrical excitation of paralyzed or weak muscles in patients with upper motor neuron lesions to produce functional movements such as sit-to-stand or walking [1]. Traditionally, during FES, skeletal muscles are activated with constant-frequency trains (CFTs), where the pulses within each train are separated by regular interpulse intervals (IPIs; Fig. 1). However, studies have shown that varying the stimulation frequency within a train markedly affects the force production from the muscle [2]. In addition, a recent study showed that varying the stimulation frequency and pattern across trains improved the muscles ability to produce 50° knee extension repetitively as compared to the performance elicited by CFTs [3]. Interestingly, Garland and Griffin [4] also showed that motor units are activated with varying patterns during volitional contraction. Hence, the stimulation patterns for optimizing force production during FES are probably complex. One way to assist the search for the optimal pattern is to use mathematical models that can predict forces accurately to a range of physiological conditions and stimulation patterns. In addition, mathematical models used in conjunction with closed loop control would enable FES systems to deliver patterns customized for each person to perform a particular task while continuously adapting the stimulation protocols to the actual needs of the patient.

Phenomenological Hill-type [5-10], Huxley-type cross-bridge[11,12], or analytical approaches [13,14] have been developed to explore different aspects of muscle contraction under both isometric and non-isometric conditions. However, each of these models either: (a) could not predict the force or motion response to a range of stimulation frequencies and patterns, (b) have a large number of free parameters that make the model identification process difficult, (c) were not tested for intact human muscles, and (d) were evaluated only under isometric conditions.

Previously, our laboratory developed isometric models for rat gastrocnemius and soleus muscles that addressed the first two shortcomings outlined above. We then extended and modified these models for human quadriceps muscles under isometric fatigue and non-fatigue conditions [15-19]. Recently, comparisons of different isometric force models to fit and predict isometric forces in response to range of stimulation trains showed that our isometric model performed better than the linear models and had similar performance when compared to Bobet-Stein's model [20,21]. Hence, for the present study, we build upon our isometric models to capture the effects of constant angular velocity (isovelocity) of the lower limb on the forces produced in response to electrical stimulation of the quadriceps femoris muscle. Modeling the isovelocity condition is important because it enables us to understand how our model behaves under the relatively simple condition of constant velocity before trying to model the more complicated non-isometric conditions, where limb velocities change as function of time. More importantly, the current model would enable us to better understand the interactions of muscle length, limb velocity, and stimulation pattern on the force produced by the muscle. This would, in turn, enable us to design stimulation patterns for FES. Hence the purposes of this study are to derive the equations to model the effect of velocity and stimulation on the muscle force under isovelocity conditions and determine if the model can capture the variations in force as a function of velocity when the muscle is activated with a range of stimulation frequencies, patterns, muscle lengths, and number of pulses.

## Methods

### Model development

The isovelocity model is based on the Hill-type isometric force model developed by our laboratory [5,16,17,22]. This isometric model is used because it is the only model that can predict forces in response to a wide range of stimulation frequencies and because the parameters in the model have a physiological basis, which make it less phenomenological than other Hill-type models. Our model divides the contractile responses of the muscle are decomposed into two distinct physiological steps: activation dynamics and the force dynamics. In addition, we developed the equations of motion for the lower limb moving at constant velocities.

### Activation dynamics

A number of complicated steps are involved between motor nerve activation by electrical stimulation and the force production by the muscle, such as release and uptake of calcium by the sarcoplasmic reticulum, binding of calcium to troponin, and the attachment of myosin filaments with actin [23]. However, Ding and colleagues [16,17,22] found that it was sufficient to model this activation dynamics through a unitless factor, *C*_*N*_, to describe the rate-limiting step before the myofilaments mechanically slide across each other and generate force. The differential equation describing this dynamics is:

(1) $$\frac{dC_{N}}{dt}=\frac{1}{\tau_{c}}\sum_{i=1}^{n}R_{i}\exp(-\frac{t-t_{i}}{\tau_{c}})-\frac{C_{N}}{\tau_{c}},$$

and whose analytical solution satisfying the initial conditions is

(2) $$C_{N}=\sum_{i=1}^{n}R_{i}(\frac{t-t_{i}}{\tau_{c}})\exp(-\frac{t-t_{i}}{\tau_{c}}).$$

where

(2a) *R*_*i *_= 1   for i = 1

(2b) *R*_*i *_= 1 + (*R*_0 _- 1)exp[-(*t*_*i *_- *t*_*i*-1_)/*τ*_*c*_]   for i > 1.

In Eqns. (1) and (2), *t *(ms) is the time since the beginning of the stimulation train, *t*_*i *_(ms) is the time of the *i*th stimulation pulse since the beginning of the stimulation train, *n *is the number of stimulation pulses before time *t *in the train, and *τ*_*c *_(ms) is the time constant controlling the transient shape of *C*_*N*_. *R*_*i *_(unitless) is the scaling term that accounts for the difference in the degree of activation by each pulse relative to the first pulse in the train [24]. The enhancement of *R*_*i *_is characterized by *R*_0 _(unitless) and its dynamics is characterized by *τ*_*c *_(ms). *R*_*i *_decays with interpulse interval *t*_*i*_-*t*_*i*-1_. Hence, *R*_*i *_= 1 for a pulse that occurs at a long time after the preceding pulse, and *R*_*i *_approaches *R*_0 _for the smallest interpulse interval tested, 5 ms.

### Force dynamics

When calcium binds to troponin, the inhibitory effect of tropomyosin is removed and results in the exposure of binding sites on actin. The crossbridges attach to actin and pull the actin filaments toward the center of the myosin filaments. The macroscopic result of this process is the shortening of the muscle and the generation of force. Force generation is modeled by a Hill-type representation of the skeletal muscle as shown in Fig. 1. Here the skeletal muscle is modeled as a spring (with stiffness *k*_*S*_), a damper (with a damping coefficient *b*), and a motor (with velocity *V*). The series spring represents the tendonous portion and the series elastic component of the muscle [25], the damper represents the viscous resistance of the contractile and connective tissue [26], and the motor represents the contractile component or the sliding of actin and myosin filaments of muscle fibers [19]. The series spring is assumed linear and the force exerted by the spring is given by

(3) >*F *= *k*_*s*_*x*,

where *k*_*S *_is the spring constant or stiffness and *x *is the displacement of the spring under the force *F*.

The damper is also assumed linear and is given by

(4) $$F=b(\dot{y}-\dot{x}),$$

where *b *is the damping coefficient, *y *is the distance moved by the right hand side of the damper in Fig. 1, and $\frac{dC_{N}}{dt}=\frac{1}{\tau_{c}}\sum_{i=1}^{n}R_{i}\exp(-\frac{t-t_{i}}{\tau_{c}})-\frac{C_{N}}{\tau_{c}},$ is the relative velocity of the damper.

The contractile velocity *V *of the motor is given by

(5) $$V=-(\dot{z}-\dot{y}),$$

where *z *is the net displacement and the negative sign accounts for the fact that the motor is shortening. All shortening contractions are taken as negative in this study. The motor, which represents the contractile element, is driven by the strongly bound cross bridges [19,27]. As there is a sigmoidal force-pCa relationship [28], Ding and coworkers [15] modeled the relationship between *V *and *C*_*N *_by a simple Michaelis-Menten term, *C*_*N*_/(*K*_*M *_+ *C*_*N*_). Hence, *V *is now given by

(6) $$V=\dot{y}-\dot{z}=B\frac{C_{N}}{K_{M}+C_{N}},$$

where *B *is the constant of proportionality and *K*_*M *_mathematically represents the sensitivity of strongly bound cross-bridges to Ca^2+^-troponin complex [22].

Differentiating Eqn. (3) with respect to time and using Eqn. (4) to eliminate $\dot{x}$ and Eqn. (6) to eliminate $\dot{y}$ gives

(7) $$\frac{dF}{dt}=k_{S}\frac{dz}{dt}+k_{S}B\;\frac{\left[C_{N}\right]}{K_{M}+\left[C_{N}\right]}-\frac{F}{\frac{b}{k_{S}}}.$$

The term *b*/*k*_*s *_represents the time constant over which the force decays. Ding and colleagues [15,22] expect the friction between actin and myosin to be higher during cross-bridge cycling due to binding between the fibers, so they set $b/k_{s}=\tau_{1}+\tau_{2}\frac{\left[C_{N}\right]}{K_{M}+\left[C_{N}\right]}$, where *τ*_1 _is the value of the time constant in the absence of bound cross-bridges and *τ*_2 _is the additional frictional component due to the cross-bridge binding. Using this for *b*/*k*_*s *_and replacing *k*_*s*_*B *with a new constant *A*, gives

(8) $$\frac{dF}{dt}=k_{S}\frac{dz}{dt}+A\frac{\left[C_{N}\right]}{K_{M}+\left[C_{N}\right]}-\frac{F}{\tau_{1}+\tau_{2}\frac{\left[C_{N}\right]}{K_{M}+\left[C_{N}\right]}}.$$

As it is experimentally difficult to measure *z *and its derivative with respect to time, *z *is viewed as a function of the knee flexion angle *θ*. Thus, *z *is written as *z *= *g*(*θ*).

Differentiating *k*_*s*_*z *with respect to time gives

(9) $$k_{S}\frac{dz}{dt}=k_{S}\frac{dg\left(\theta \right)}{d\theta }\frac{d\theta }{dt}=G(\theta )\dot{\theta },$$

where $\dot{\theta }$ = *dθ*/*dt*, is the angular velocity of the limb. Substituting Eqn. (9) into Eqn. (8) gives

(10) $$\frac{dF}{dt}=G(\theta )\dot{\theta }+A\frac{\left[C_{N}\right]}{K_{M}+\left[C_{N}\right]}-\frac{F}{\tau_{1}+\tau_{2}\frac{\left[C_{N}\right]}{K_{M}+\left[C_{N}\right]}}.$$

When $\dot{\theta }$ = 0, the above equation reduces to the isometric form explored in previous studies [16,17,22]. By assuming only *A *to be a function of the knee flexion angle *θ*, and by fixing other parameter at their 40° knee flexion angle values, the isometric form of the model is able to capture changes in force with muscle length. *A *was found to vary in a parabolic manner and was modeled as

(11) *A*(*θ*) = *a*(40 - *θ*)^2 ^+ *b*(40 - *θ*) + *A*_40_,

where *A*_40 _is the value of *A *at 40° of knee flexion, and *a *and *b *are constants that need to be identified for each subject [18]. Hence, *A *captures the effect of muscle length on the force due to stimulation and the model is able to predict the force response to a wide variety of stimulation frequencies.

It is necessary to identify the functional form of *G*(*θ*) to model the variation of force with velocity. As seen from Eqn. (9), *G*(*θ*) is dependent on an unknown function g(*θ*) and *k*_*S*_. Previous studies [29,30] have used exponential functions to model the nonlinear relationship between knee flexion angle and joint stiffness torque. Hence, we assumed *G*(*θ*) to be of the form

(12) *G*(*θ*) = *V*_1_*θ *exp(-*V*_2_*θ*),

where *V*_1 _and *V*_2 _are constants to be identified for each subject. In addition, Heckman and colleagues [31] and de Haan [32] showed that in cat and rat medial gastrocnemius muscle, the force-velocity relation was affected by stimulation frequency. Hence, to account for the coupling between force, velocity, and activation in our modeling we multiplied *G*(*θ*)$\dot{\theta }$ by the Michaelis-Menten term *C*_*N*_/(*K*_*M *_+ *C*_*N*_). Considering the above assumption and the functional form of *A*(*θ*), Eqn. 10 can be written as

(13) $$\frac{dF}{dt}=\left[V_{1}\theta \exp(-V_{2}\theta )\dot{\theta }+a{\left(40-\theta \right)}^{2}+b(40-\theta )+A_{40}\right]\frac{\left[C_{N}\right]}{K_{M}+\left[C_{N}\right]}-\frac{F}{\tau_{1}+\tau_{2}\frac{\left[C_{N}\right]}{K_{M}+\left[C_{N}\right]}}.$$

Eqns. (2) and (13) represent the complete set of equations for this study. In addition, the following constraints were imposed during estimation of model parameters and prediction of experimental forces for isovelocity movements: (1) *θ *≥ 0, (2) *A*(*θ*) ≥ 0, and (3) *F *≥ 0. The first constraint comes from the fact that we consider the motion of the leg between 90° to 0° of knee flexion. The second and third constraints were imposed to ensure that the force during stimulation is never negative. Eqns. (2) and (13) model the forces due to stimulation of the muscle and are governed by ten parameters: *R*_0_, τ_*c*_, *a*, *b, A*_40_, *τ*_1_, *τ*_2_, *K*_*M*_, *V*_1_, and *V*_2 _(see Table 1).

**Table 1:** Definition of symbols used in the model.

Symbol	Unit	Definition
*C*_*N*_	---	normalized amount of Ca^2+^-troponin complex
*t*	ms	time since the beginning of the stimulation
*t*_*i*_	ms	time when the *i*th pulse is delivered
*τ*_*c*_	ms	time constant controlling the rise and decay of *C*_*N*_
*R*_0_	----	term characterizing the magnitude of enhancement in *C*_*N *_from the following stimuli
*F*	N	instantaneous force due to stimulation
*k*_*s*_	N/m	spring stiffness
*b*	Ns/m	damping coefficient
*V*	m/s	shortening velocity of motor
*A*_40_	N/ms	scaling factor for force at 40° of knee flexion
*a*	N/ms-deg^2^	scaling factor to account for force at each knee flexion angle
*b*	N/ms-deg	scaling factor to account for force at each knee flexion angle
*θ*	deg	knee flexion angle
*H*	Nm	resistance moment knee extension
*l*	m	distance between knee center of rotation and center of mass of leg
*L*	M	length of lever arm from center of force transducer to center of knee rotation
*V*_1_	N/deg^2^	scaling factor in the term *G*(*θ*)
*T*_*STIM*_	Nm	knee joint torque due to stimulation
*mg*	N	weight of the tibia and foot
*V*_2_	1/deg	constant that is linearly realted to *τ*_2 _(see Eqn. 20)
*K*_*m*_	---	sensitivity of strongly bound cross-bridges to *C*_*N*_
*τ*_1_	ms	time constant of force decline in the absence of strongly bound cross-bridges
*τ*_2_	ms	time constant of force decline due to the extra friction between actin and myosin resulting from the presence of strongly bound cross-bridges
*M*	N	resistance to knee extension
*F*_*EXT*_	N	experimental force measured by the KinCom dynamometer

It is important to understand the practical meaning of *F *in Eqn. (13). The model must be fitted to experimental force data to evaluate the parameters (see SectionB.5). The experimental force is measured in a Kin-Com machine by placing a force transducer above the ankle joint (see **Equipment and experimental setup **section). When the quadriceps femoris muscle is stimulated, it exerts a force on the patellar ligament, which then transfers the quadriceps force onto the tibia in a complicated manner [33]. Hence, the quadriceps muscle exerts a force, *F*, on the transducer placed above the ankle joint. This force *F *is a function of patellar tendon force and the distance from the center of the force transducer to the center of knee rotation. Hence, the *F *in Eqn. (13) is now the force above the ankle joint exerted by the quadriceps in response to stimulations through the knee joint. From here on, we define this force (*F*) as the force due to the stimulation, as we have done previously [15,18], so that the parameters incorporate the kinematic transfer of force from the muscle to the transducer.

### Equations of motion

**Figure 2:**
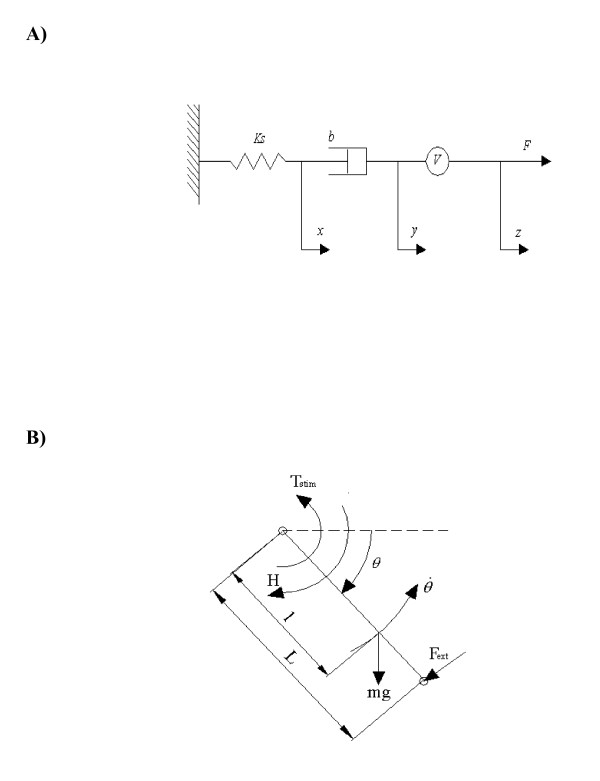
A) Schematic representation of a Hill-type model used for modeling the muscle's response to electrical stimulation. The muscle modeled as a linear series spring, linear damper, and a motor. The parallel elastic element was neglected because for the range of motion studied in the current study the passive forces are smaller than the active force. *k*_*s *_is the spring constant of the series element, *b *is the damping coefficient of the damper, and *V *is the velocity of the motor. The force exerted by the spring and damper are *k*_*s*_*x *and $b(\dot{y}-\dot{x})$, respectively. The velocity of the motor is given by $V=\dot{y}-\dot{z}=B\frac{C_{N}}{K_{M}+C_{N}}$, where *B *is the constant of proportionality (see text for details). B) Schematic representation of the leg modeled as single rigid body segment (tibia) when subjected to stimulation under isovelocity conditions. In the isovelocity mode, the KinCom arm moves the tibia at a constant angular velocity ($\dot{\theta }$ = constant). *θ *is the knee flexion angle. L is the distance from the knee joint center to the center of the force transducer placed above the ankle and *l *is the distance from the knee center of rotation to the center of mass of the tibia. *T*_*stim *_is the torque due to stimulation, *F*_*EXT *_is the force measured by the KinCom dynamometer, *mg *is the weight of the tibia-foot complex (foot not shown in figure), and *H *is the resistance moment to knee extension due to visco-elasticity of the musculotendon complex of the knee joint.

Fig. 2B shows a schematic representation of the leg when the tibia is moving at a constant angular velocity, with the stimulations being applied to the quadriceps femoris muscle. The instantaneous moment dynamic equation about the center of knee rotation when the tibia is moving at constant angular velocity is:

(14) *F*_*EXT*_*L *- *T*_*STIM *_+ *mg *cos*θ*·*l *+ *H *= 0,

where *F*_*EXT *_is the resistance the Kin-Com exerts above the ankle joint to move the tibia with a constant angular velocity and is the measured force from the Kin-Com,*T*_*STIM *_is the torque at the knee joint due to stimulation of the quadriceps femoris muscle, *mg *is the weight of the tibia and foot, *H *is the resistance moment to knee extension due to visco-elasticity of the musculotendon complex of the knee joint, *θ *is the knee flexion angle, *l *is the distance between knee center of rotation and center of mass of the leg below the knee, and *L *is the length of the lever arm from the center of the force transducer above the ankle joint to the center of knee rotation. The right hand side of Eqn. (14) is zero because there is no angular acceleration during the isovelocity phase of the contraction. Because the experimental force is measured with a force transducer placed just above the ankle joint we can write

(15) *T*_*STIM *_= *F*·*L*,

where *F *and *L *are as defined before. Substituting Eqn. (15) into Eqn. (14) and rearranging we get

(16) $$F_{EXT}=F-mg\cos\theta \cdot \frac{l}{L}-\frac{H}{L}.$$

To model the resistance to knee extension, *H*, due to visco-elasticity of the musculotendon complex of the knee joint, it is necessary to consider stiffness and damping factors, which are functions of knee flexion angle and angular velocity, respectively [13,34]. These functions are complicated and nonlinear [29,34,35]. Preliminary passive force measurements on healthy subjects, where the knee was extended at a constant velocity, showed that *H*/*L *= *R *cos(*θ*) well represented the measured data. *R *was found to be independent of *θ *or $\dot{\theta }$ for healthy subjects, which may not be the case of spinal cord injured and stroke patients, where other passive factors like spasticity play an important rule. The above form of *H*/*L *simplifies equation 16 to

(17) $$F_{EXT}=F-(mg\cdot \frac{l}{L}+R)\cos\theta .$$

Replacing $\left(mg\cdot \frac{l}{L}+R\right)$ by *M *in Eqn. (17) we obtain,

(18) *F*_*EXT *_= *F *- *M *cos*θ*.

Thus, to obtain muscle force due to stimulation, *F*, it is necessary to add *M *cos*θ *to *F*_*EXT*_, the force measured by the Kin-Com force transducer. This was done during data analysis (see **Experimental procedure for model development **section for details), so that experimental forces can be compared to model predictions.

### Subjects

**Figure 3:**
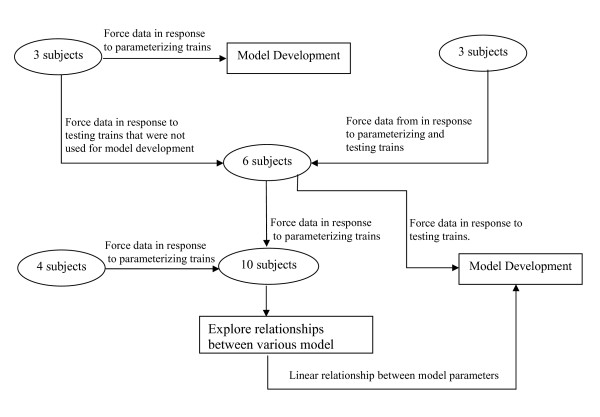
Overview of the distribution of subjects used for model development and validation. See text for details about the characteristics of the parameterizing and testing trains.

Ten healthy subjects (5 women and 5 men with a BMI ≤ 32) ranging in age 18 to 35 years were recruited for this study (see Fig. 3). Data collected from three subjects were used to develop the form of the model. Data from these three subjects and three additional subjects that were not used for model development, were used to validate the model (Fig. 3). In an effort to simplify the model, linear correlations between different model parameters determined for the six subjects tested. However, because these relationships were inconclusive, we tested four additional subjects (Fig. 3). Before testing, each subject signed an informed consent form approved by the University of Delaware Human Subject Review Board. All the subjects recruited for the study were acclimatized to electrical stimulation as they have previously participated in studies that involved electrical stimulation.

### Equipment and experimental setup

Subjects were seated on a computer controlled (KinCom III 500-11, Chattecx Corporation, Chattanooga, TN) dynamometer with their hips flexed to ~75° [36]. The dynamometer axis was aligned with the knee joint axis and the force transducer pad was positioned anteriorly against the tibia, 4 cm proximal to the lateral malleolus. Two 7.62 cm × 12.7 cm self-adhesive electrodes were used to stimulate the muscle. With the knee positioned at 90°, the anode was placed proximally over the motor point of the rectus femoris portion of the quadriceps femoris muscle. The cathode was placed distally over the vastus medialis motor point with the knee in 15° of flexion to compensate for skin movement during knee extension [37]. The trunk, pelvis, and thigh of the leg being tested were each stabilized with inelastic straps. A Grass S8800 stimulator with an SIU8T stimulus isolation unit (Grass Instruments, West Warwick, RI) was used for stimulation. The stimulator was driven by a personal computer using customized LabView (National Instruments, Austin, TX) software. Force and motion data from the transducer were sampled at 200 Hz using an analog-to-digital board. The data were then analyzed using a custom program written in LabView.

Using a KinCom dynamometer, isometric and isovelocity force data were collected from the human quadriceps femoris muscle in response to electrical stimulation. Each subjects performed a maximum voluntary isometric contraction (MVIC) of the quadriceps femoris muscle with the knee positioned at 90° of flexion. The burst-superimposition technique was used to ensure that a true maximal contraction was being performed [38]. Next, with the knee at 90° flexion the stimulation amplitude was set to activate ~20% of the muscle MVIC using a 300 ms-long 100-Hz stimulation train. Once the amplitude was set, it was held constant for the remainder of the session. The pulse duration was fixed at 600 μs throughout this study. To ensure consistency in the force responses to stimulation, we first potentiated the muscle using 14-Hz, 770 ms long trains before delivering the parameterizing and testing trains (see the section below for details of the parameterizing and testing trains).

### Experimental procedure for model development

First, three subjects were recruited to participate in two testing sessions. A 48-hour rest period separated the two sessions. During the first session, testing was performed isometrically at angles of 15°, 40°, 65°, and 90°. The order of testing for the four angles was randomly determined and five minutes of rest was provided between each angle. Five minutes following the isometric testing, subjects were tested at one of the four isovelocity speeds of -25°/s, -75°/s, -125°/s, or -200°/s (all shortening velocities are assigned negative values in this study). During the second session, subjects were tested at the remaining three velocities. The order of testing the three velocities was randomly determined and five minutes of rest was provided between each velocity.

For the isometric testing, two one-second long trains were used to stimulate the muscle. Each train had an initial interpulse interval (IPI) of 5 ms and the remaining IPIs were either 20 or 80 ms (Fig. 1). These two variable-frequency trains (VFTs) were referred to as VFT20 and VFT80, respectively. Previous study by Ding and colleagues [5] showed that our model had the best predictive ability for human quadriceps femoris muscle if the model's parameter values were identified using force responses to these two trains. Within the stimulation protocol, first the VFT80 train followed the VFT20 train and then these trains were delivered in reverse order. Only one train was delivered every 10 s to minimize muscle fatigue. For the isovelocity study, 16 different trains were used: six constant-frequency trains (CFTs) referred to as CFT10, CFT20, CFT30, CFT50, CFT70, and CFT100; six VFTs referred to as VFT20, VFT30, VFT50, VFT70, VFT80, and VFT100; and four doublet frequency trains (DFTs) with 5 ms doublets throughout the train referred to as DFT30, DFT50, DFT70, and DFT100 (Fig. 1). The maximum number of pulses in each train was limited to 50, except for VFT20, which had a maximum of 51 pulses.

During isovelocity testing the KinCom was set to the Isokinetic mode, where the subjects remained passive and the KinCom arm moved the leg at predetermined speeds. The leg motion was initiated at 110° of knee flexion and stimulation began when the leg reached 90° of knee flexion and was terminated at 15° of knee flexion, unless all the pulses were already delivered. The KinCom arm moved the leg to 0° of knee flexion and then returned the leg back to 110° of knee flexion at a constant velocity of 25°/s. A 10 s rest time was provided before delivering the next train. Software, custom written in LabView, was used to determine the timing of each of the pulses delivered to each subject. In addition, force data were collected while passively moving the leg at constant velocity of -25°/s, -75°/s, -125°/s, and -200°/s from 110° to 0° of knee flexion to determine the value of *M*. The absolute value of *M *cos*θ *was then added to the measured force data, *F*_*EXT*_, to obtain the stimulation muscle-joint force, *F *(Eqn. 18) throughout the study.

Parameter identification for model development: Based on our model derivation, the term *G*(*θ*)$\dot{\theta }$ explicitly modeled the effect of velocity on the force produced by the muscle. In turn, *G*(*θ*)$\dot{\theta }$ is characterized by the parameters *V*_1 _and *V*_2_. Hence, all the isometric parameters *a*, *b*, *A*_40_, *τ*_1_, *τ*_2_, and *K*_*M *_were assumed to be constant for isovelocity conditions and only parameters *V*_1 _and *V*_2 _were identified under isovelocity conditions. Under isometric conditions the angular velocity, $\dot{\theta }$, is zero hence Eqn. (13) reduces to

(19) $$\frac{dF}{dt}=\left[a{\left(40-\theta \right)}^{2}+b(40-\theta )+A_{40}\right]\frac{\left[C_{N}\right]}{K_{M}+\left[C_{N}\right]}-\frac{F}{\tau_{1}+\tau_{2}\frac{\left[C_{N}\right]}{K_{M}+\left[C_{N}\right]}}.$$

**Figure 4:**
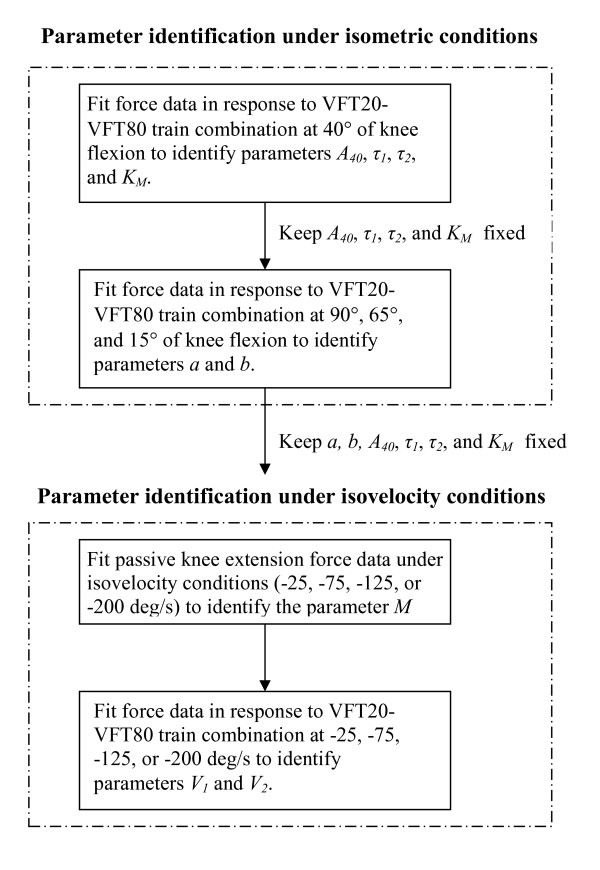
Flowchart of the steps involved in the parameter identification during the model development phase. Please note, during the model validation phase the steps involved in the parameter identification are identical to those outlined in the above flow chart, except that parameters *M*, *V*_1_, and *V*_2 _will be identified at the velocity determined from the model development phase.

Ding and colleagues [16,17] have shown that a fixed value of 20 ms for τ_*c *_and 2 for *R*_0 _are sufficient for human quadriceps muscles under non-fatigue condition. Based on the results of our previous study the values *A*_40_, *τ*_1_, *τ*_2_, and, *K*_*M *_[18] are identified first at 40° of knee flexion by fitting Eqns. (1) and (12) to the forces produced by stimulating the muscle with a combination of VFT20 and VFT80 trains (Fig. 4). These parameter values were then kept fixed and the values of *a *and *b *were identified at knee flexion angles of 15°, 65°, and 90° by fitting the measured force response to the VFT20-VFT80 train combination. The values of *a *and *b *were obtained by first determining the value of *A *from fitting the VFT20-VFT80 force responses at angles of 15°, 65°, and 90° [18] and then fitting the values of *A *at the above four angles to the parabolic equation given by *a*(40 - *θ*)^2 ^+ *b*(40 - *θ*) + *A*_40_. Fitting of measured and modeled data was carried out using a derivate based optimization technique in MATLAB.

Under isovelocity conditions, first the value of *M *was obtained by fitting the function *M *cos*θ *to the passive knee extension force data from 90° to 15° of knee flexion at each of the four velocities. The absolute value of *M *cos*θ *was then added to the measured force data, *F*_*EXT*_, to obtain the stimulation muscle-joint force, *F *(Eqn. 18). The model (Eqns. 1 and 13) was then fitted to the forces elicited by the VFT20-VFT80 train combination at -25°/s, -75°/s, -125°/s, and -200°/s to obtain the values of *V*_1 _and *V*_2 _at each of four velocities. This was done to determine the best velocity to identify the values of *V*_1 _and *V*_2_. For all the data collected, the two occurrences of each of the stimulation trains were averaged to reduce the effects of physiological variability on the muscle's response to each train.

### Results for model development

**Figure 5:**
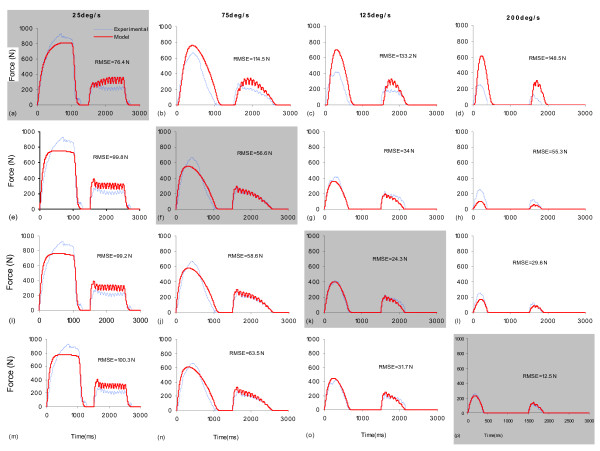
Experimental, fitted, and predicted forces in response to the VFT20-VFT80 train combination from a typical subject at different shortening velocities during the model development phase. For each row, the shaded panel represents the fittings of the VFT20-VFT80 experimental data to that velocity, while the non-shaded panels represent model predictions to the other three velocities. For example, the shaded panel (f) at -75°/s represents the fitting of the isovelocity model to the experimental forces data, while the non-shaded panels (e), (g), and (h) are the predictions of the model at -25°/s, -125°/s, and -200°/s, respectively. These data were used to determine the best velocity to identify the parameters *V*_1 _and *V*_2_. RMS Force (N) errors between measured and modeled force data in response to the VFT20-VFT80 train are presented in each panel. It should be noted that the experimental data in the figure are means of two trails.

From Fig. 5 we see that -200°/s is the only velocity that the model both fits the VFT20-VFT80 force data at its own velocity well (i.e., -200°/s, Fig. 5p), and predicts the VFT20-VFT80 force data at each of the other three velocities very well (Figs. 5m, 5n, 5o). Similar results are observed for the other two subjects. Hence, for the model development and validation stages, the VFT20-VFT80 train combination at -200°/s is used to identify the values of *V*_1 _and *V*_2_. In addition, because the values of *M *at different velocities are generally within ten percent of each other, the value of *M *at -200°/s is used to correct all isovelocity measured data (See Table 2).

**Table 2:** Values of parameter *M *at each velocity for the three subjects tested.

Velocity (°/s)	Subject 1	Subject 2	Subject 3
-25	-65.8	-119.6	-58.0
-75	-62.7	-110.4	-46.5
-125	-68.9	-154.2	-71.2
-200	-66.5	-120.0	-52.9

### Validation of the model

The model was validated by determining its ability to predict forces in response to wide range stimulation frequencies and patterns at velocities of -25°/s, -75°/s, -125°/s, and -200°/s. Data were collected from three additional subjects. The same protocol used for the first three subjects recruited for the model development phase was tested and the data for the six subjects were pooled (Fig. 3).

### Data analysis for model validation

% error, linear regression trend lines, and paired t-tests were used to test how well the model predicted the experimental forces. Mean % errors between the model and experimental forces normalized to the experimental peak force and measured at each 5 ms time interval were calculated for each subject. The experimentally measured and model's predicted force-time integrals and peak forces were averaged across six subjects at each velocity and at each stimulation pattern. Paired *t*-tests were used to compare the average measured and predicted data. The paired t-test comparisons were considered significant if p = 0.05. Linear regression trend lines were used to determine how well the model predicted the force-time integrals (area under the force-time plots) and the peak forces for each train tested at each velocity for all the six subjects. The slope of the trend line was set to one and the intercept was set to zero. A perfectly accurate model would have a coefficient of determination, *R*^2^, of one.

### Model simplification

Because a model with fewer parameters has better predictive abilities and is desired by designers of the FES systems [39], we have tried to limit the number of free parameters for model. We therefore determined the linear correlations between parameters *V*_1 _and *V*_2 _and each of the other model parameters for the six subjects tested, i.e., *V*_1_-vs-*a*, *V*_1_-vs-*b*, *V*_1_-vs-*A*_40_, *V*_1_-vs-*K*_*M*_, *V*_1_-vs-*τ*_1_, *V*_1_-vs-*τ*_2_, *V*_2_-vs-*a*, *V*_2_-vs-*b*, *V*_2_-vs-*A*_40_, *V*_2_-vs-*K*_*M*_, *V*_2_-vs-*τ*_1_, and *V*_2_-vs-*τ*_2_. However, because these relationships were inconclusive, we tested four more subjects (see Fig. 3). Each of the four subjects participated in one testing session. The procedure for testing them was similar to that described above, except that these four subjects were tested only with VFT20 and VFT80 trains under isometric conditions of 15°, 40°, 65°, and 90° of knee flexion and at the isovelocity speed of -200°/s. The values of each of the parameters (*a*, *b, A*_40_, *τ*_1_, *τ*_2_, *K*_*M*_, *V*_1_, and *V*_2_) were then obtained as described above. For each of the above relationships we fitted the data with a linear trend-line and calculated the R^2 ^values for all the 10 subjects tested. R^2 ^values for the relationships of parameters *V*_1 _and *V*_2 _with the other model parameters showed that only parameters *b *and *τ*_2 _had a high correlation to *V*_2 _with R^2 ^values of 0.62 and 0.81 (p = 0.00035), respectively (see Table 3). Because the correlation between *V*_2 _and *τ*_2 _was greater than the correlation between *V*_2_and *b*, we adopted the relationship between *V*_2 _and *τ*_2 _that was given by

**Table 3:** R^2 ^values for the relationships of parameters *V*_1 _and *V*_2 _with the other model parameters

	*V*_1_-vs-*a*	*V*_1_-vs-*b*	*V*_1_-vs-*A*_40_	*V*_1_-vs-*K*_*M*_	*V*_1_-vs-*τ*_1_	*V*_1_-vs-*τ*_2_	*V*_2_-vs-*a*	*V*_2_-vs-*b*	*V*_2_-vs-*A*_40_	*V*_2_-vs-*K*_*M*_	*V*_2_-vs-*τ*_1_	*V*_2_-vs-*τ*_2_
R^2^	0.05	0.00	0.04	0.27	0.00	0.24	0.46	0.62	0.04	0.02	0.16	**0.81**

(20) *V*_2 _= 0.0002 * *τ*_2 _+ 0.0048.

The above empirical relationship between *V*_2 _and *τ*_2 _was then incorporated in the model and the force responses to the VFT20-VFT80 train combination at -200°/s were fit again to calculate the new value of *V*_1 _for the six subjects used to validate the model.

### Sensitivity analysis

Sensitivity analysis was performed using Fourier Amplitude Sensitivity Test (FAST) to obtain a measure of the sensitivity of our model's output to changes in model parameters. The FAST method was used to estimate the expected value and variance of the output, and the contribution of individual inputs to the variance of the output [40]. The ratio of the contribution of each input to the total output variance is referred to as the first order sensitivity index and can be used to rank the inputs [41]. For the current sensitivity analysis, the output of the model was the force-time integral (area under the force-time curve) in response to a 50-pulse, 33-Hz stimulation train at each of the four velocities tested and under isometric conditions at 90° of knee flexion. Parameters *τ*_*c *_and *R*_0 _were kept fixed at 20-ms and 2, respectively, and equation 20 was used to calculate the value of *V*_2_. Values of the other seven parameters were varied within the following ranges: *a *(-0.003 to -0.0005), *b *(-0.22 to -0.02), *A*_40 _(1.5 to 8.7), *τ*_1 _(26 to 76), *τ*_2 _(58 to 280), *K*_*M *_(0.15 to 0.66), and *V*_1 _(0.0007 to 0.0028). The range of values for the above seven parameters were determined based on the parameter values of 10 subjects in the current study. SIMLAB [42] software was used to carry out the sensitivity analysis. A total of 623 sample sets were generated using Monte Carlo methods. Each sample set consisted of the different values of the eight model parameters. FAST first order sensitivity index was calculated for each parameter. The higher the value of the sensitivity index of a parameter, the greater is the sensitivity of the model output (force-time integral) to changes in that model parameter.

**Figure 6:**
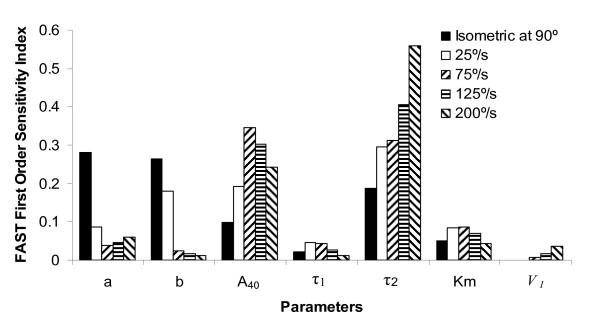
Bar graphs of FAST first order sensitivity index of the seven model parameters under isometric conditions at a knee flexion angle of 90° and the four isovelocity conditions of 25°/s, 75°/s, 125°/s, and 200°/s. Higher the value of the sensitivity index of a parameter, greater is sensitivity of the model output (force-time integral) to changes in that model parameter.

Sensitivity analysis showed that under isometric conditions at 90° of knee flexion, parameters *a*, *b*, *A*_40_, and *τ*_2 _accounted for ~85% of the total variation of the force-time integral (Fig. 6). Also, parameter *V*_1 _had no effect on the output under the isometric condition. This result is not surprising as parameter *V*_1 _accounts for the effects velocity. At faster speeds the output variance is dominated by parameters *A*_40 _and *τ*_2 _(Fig. 6). For example, at a shortening speed of 200°/s parameters *A*_40 _and *τ*_2 _account for ~25% and ~56% of the output variance, respectively. In addition, with increase in shortening velocity from 0°/s to 200°/s, the percentage of the output variance accounted by parameter *τ*_2 _increased from 18.74% at 0°/s to 55.94% at 200°/s (Fig. 6). This is because parameter *τ*_2 _is related to parameter *V*_2 _through equation 20 and with increasing shortening velocities the effects of parameter *V*_2 _on the output of the model increases. At all the five speed conditions tested, the contributions of parameters *τ*_1_, *K*_*M*_, and *V*_1 _to the output variance was small (Fig. 6).

## Results

**Figure 7:**
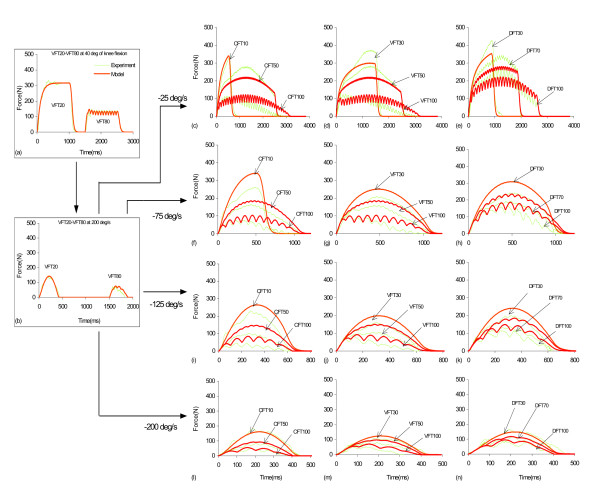
Experimental, fitted, and predicted forces from a typical subject's quadriceps femoris muscle at different constant shortening speeds during the model validation phase. (a): Fitting of the experimental force data in response to VFT20-VFT80 train combination at 40° of knee flexion (isometric) to determine the values of *a*, *b*, *A*_40_, *τ*_1_, *τ*_2_, and *K*_*m *_(b): Fitting the experimental force data in response to VFT20-VFT80 train combination at 200°/s to determine the value of *V*_1_. (c)-(e): Comparison of experimental and predicted forces in response to CFT10, CFT50, and CFT100 (panel c), VFT30, VFT50, and VFT100 (panel d), and DFT30, DFT70, and DFT100 (panel e) at a shortening speed of -25°/s. (f)-(n): Comparison of experimental and predicted forces in response to the above stimulation trains at -75°/s (panels f-h), -125°/s (panels i-k), and -200°/s (panels l-n). The isovelocity model parameter values for this subject were: *a *= -0.00165, *b *= -0.0662, *A*_40 _= 3.42686, *τ*_1 _= 30.69527, *τ*_2 _= 91.07621, *K*_*m *_= 0.23564, *R*_0 _= 2, *τ*_*c *_= 20, and *V*_1 _= 0.00074.

Predicted and experimental force data in response to simulation from a typical subject are shown in Fig 7. The model was first parameterized under isometric and isovelocity conditions to identify the model parameters (Fig. 7a and 7b). Then, the model predicted the force response to wide range of stimulation patterns and inter-pulse intervals at the four velocities tested (Fig. 7c–k). Percentage RMS errors between modeled and experimental forces determined for each subject at each stimulation pattern and velocity showed that the errors were in general less than 20% (Table 4).

**Table 4:** Mean % errors (± SE) between the model and experimental forces normalized to the experimental peak force and measured at each 5 ms time interval for each subject. Data are the averages (± SE) across 6 IPIs (CFTs or VFTs) or 4 IPIs (DFTs) tested.

	**-25°/s**			**-75°/s**			**-125°/s**			**-200°/s**		
%Error	CFT	VFT	DFT	CFT	VFT	DFT	CFT	VFT	DFT	CFT	VFT	DFT

S1	8.2 (1.3)	9.7 (1.2)	10.7 (0.5)	16.7 (0.6)	17.4 (1.2)	16.8 (0.4)	18.8 (4.9)	18.7 (3.4)	11.9 (1.4)	11.6 (0.8)	9 (0.9)	8.6 (1.0)
S2	12.6 (1.6)	13.9 (1.8)	12.4 (1.8)	11.9 (1.8)	10.9 (1.0)	10 (0.5)	16.9 (3.6)	14.9 (2.7)	11.3 (0.7)	18.8 (2.9)	10.1(1.6)	9.5 (2.7)
S3	18.3 (5.3)	17.6 (3.2)	13.5 (3.2)	11.7 (2.1)	10.9 (0.6)	11 (0.6)	12.6 (3.2)	10.6 (1.2)	11(2.5)	20.1(5.4)	12.2(2.0)	11.6(2.4)
S4	11.9 (2.7)	11.2 (2.3)	9.3 (0.8)	21.2 (2.8)	21.5 (1.8)	20.3 (1.0)	35.5 (7.2)	29.1(4.3)	25.1(1.8)	19.2(6.9)	17 (3.1)	10.0(1.2)
S5	24.0 (8.0)	16.5 (4.9)	18.6 (7.8)	17.2 (4.7)	10.9 (0.7)	11.4 (2.7)	17.5 (2.3)	17.6 (2.8)	16.3(1.1)	27.2(3.9)	22.5(1.1)	22.4 (2.0)
S6	9.3 (.14)	8.7 (1.7)	6.9 (1.7)	22.8 (1.9)	20.2 (1.8)	22.5 (1.6)	29.5 (1.7)	23.5 (1.7)	23.6(1.2)	12.3(1.5)	11.4(0.8)	11.0 (1.3)

**Mean**	14.1 (3.4)	13.0 (2.5)	11.9 (2.6)	16.9 (2.3)	15.3 (1.2)	15.4 (1.1)	21.8 (3.8)	19.1 (2.7)	16.6 (1.5)	18.2 (2.6)	13.7 (1.6)	12.2 (1.8)

**Figure 8:**
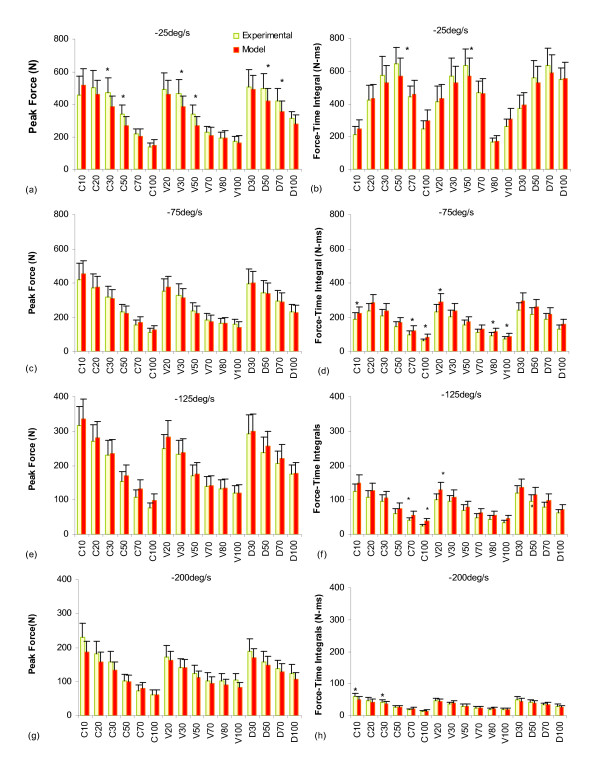
Bar graphs of comparisons between the mean (+ standard error) experimental and predicted peak forces (a, c, e, and g) and force-time integrals (b, d, f, and h) of six subjects used for validation of the isovelocity model. Responses to CFTs with interpulse intervals of 10, 30, 50, 70, and 100 ms (C10, C30, C50, C70, C100), responses to VFTs with interpulse intervals of 20, 30, 50, 70, 80, 100 ms (V20, V30, V50, V70, V80, and V100), and responses to DFTs with interpulse intervals of 30, 50, 70, 100 ms (D30, D50, D70, and D100) at shortening velocities of -25°/s (a and b), -75°/s (c and d), -125°/s (e and f), and -200°/s (g and h) are shown. * = *p *≤ 0.05 (see text for details). It should be noted that the experimental data in the figure are means of two trails. Also, note the difference in scaling of the y-axis between panels a, b, c, and d and panels e, f, g, and h.

At -75°/s, -125°/s, and -200°/s there were no significant differences between the measured and predicted peak forces for each of the IPIs and patterns tested (Figs. 8c, 8e, and 8g). In contrast, at -25°/s the model underestimated the peak forces at IPIs of 30 and 50 ms for both the CFTs and VFTs and at IPIs of 50 and 70 ms for the DFTs (Fig. 8a). For the force-time integrals, at -25°/s there were no significant differences between the measured and predicted force-time integrals for any of the IPIs and patterns tested. In contrast, at -75°/s and -125°/s the model overestimated the force-time integrals for several IPIs of the CFTs, VFTs, and DFTs (see Figs. 8d and 8f). At -200°/s, however, the model underestimated the force-time integrals for CFT10 and CFT30 (Fig. 8h).

**Figure 9:**
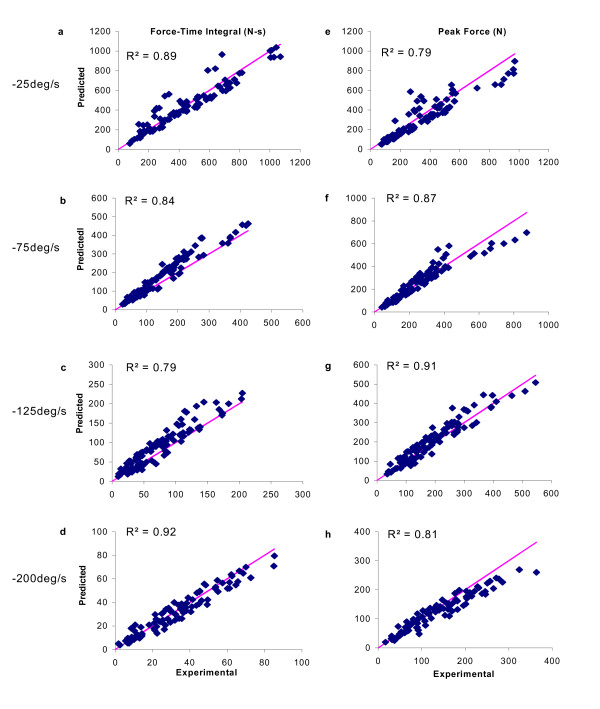
Plots of force-time integrals (a, c, e, and g) and peak forces (b, d, f, and h) of experimental versus predicted forces for six subjects at shortening velocities of -25°/s (a and b), -75°/s (c and d), -125°/s (e and f), and -200°/s (g and h). Solid lines in (a)-(e) are the identity lines. R^2 ^values were calculated.

In general, the model predicted the IPI for each pattern that produced the maximum force-time integrals and the maximum peak forces (Fig 8). For example, at -25°/s the maximum force-time integral for both predicted and measured data occurred at an IPI of 50 ms for the CFT, 50 ms for the VFT, and 70 ms for the DFT. The coefficients of determination between the measured and predicted forces showed that the model accounted for ~86% and ~85% (average values for the four velocities tested) of the variances in the measured force-time integrals and peak forces, respectively (Fig. 9).

## Discussion

In this study we developed a mathematical model of healthy human quadriceps femoris muscle that predicted forces under isovelocity conditions. Our results showed that our model had the ability to predict the force responses of the quadriceps femoris muscle to a wide range of clinically relevant stimulation frequencies and patterns when the leg was moved at a variety of constant velocities. In the current model, by identifying the values of the parameters *a*, *b, A*_40_, *τ*_1_, *τ*_2_, and *K*_*M *_under isometric conditions, only the value of the parameter *V*_1 _needed to be identified at -200°/s for the model to capture the shortening and lengthening forces of the muscle over a wide range of constant velocities. All the above parameters were identified by fitting the force responses to only two trains, the VFT20-VFT80 train combination.

The term *G*(*θ*)$\dot{\theta }$ (see Eqn. (12)) was motivated by the formulation that represents the muscle by a motor-damper-spring combination in series. Other than this new term, the current model used the same equations used for the isometric models that we developed previously [16-18]. In isometric contractions, the motor's rate of shortening is balanced by the damper and spring to produce muscle force. For shortening contractions, however, the shortening of the muscle reduces stretching of the damper and spring, resulting in lower muscle forces. Physiologically, because the cross-bridge recycling rate, which is modeled as the motor's rate of shortening, is finite, increased rates of shortening reduce the stretching of the muscle's viscous and elastic components, resulting in lower muscle forces. The exact form of the function *G*(*θ*)$\dot{\theta }$ = *V*_1_*θ *exp(-*V*_2_*θ*)$\dot{\theta }$ captures the above non-isometric effects and accounts for the influence of the knee joint kinematics on the force-velocity relationship. Interestingly, a correlation was found between the parameters *V*_2 _and *τ*_2 _(see Eqn. (20)). Wexler and colleagues [19] have shown that *τ*_2 _varied in muscles with different fiber type composition. Previous studies have shown that fiber type composition plays an important role in influencing the force-velocity relationship [43,44]. The influence of fiber type on the force-velocity relationship in the current model was captured through the parameter *V*_2_, which was incorporated in the function *G*(*θ*)$\dot{\theta }$ = *V*_1_*θ *exp(-*V*_2_*θ*)$\dot{\theta }$; the relationships between model parameters and muscle type were supported by the *τ*_2_-*V*_2 _relationship.

**Figure 10:**
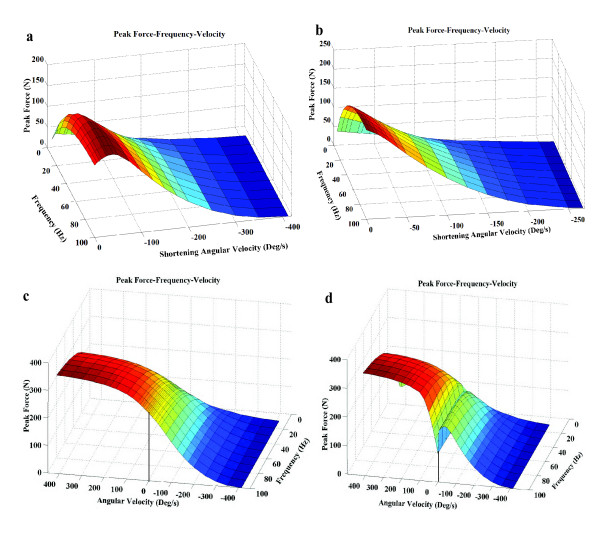
Variations in the force velocity relationship with stimulation frequency, number of pulses, and the length where the stimulation is initiated. (a) The modeled peak force versus velocity curve does not follow the hyperbolic relationship when the stimulation train is initiated at 90° of knee flexion and is terminated when either 15° of knee flexion is reached or when 50 pulses were delivered. (b) Change in force velocity curve when the stimulation train is initiated at a knee flexion angle of 60°, the optimal muscle length for this subject. (c) Shortening contractions (negative angular velocities) and lengthening contractions (positive angular velocities) were performed when the leg was moving between 90° to 15° of knee flexion and the stimulation was applied throughout the range of motion. The model accurately captures the shape during shortening and lengthening contractions. The isomeric peak force was plotted at 60° of knee flexion. (d) The plot when the leg moves through the same shortening range (90° to 15°) and lengthening range (15° to 90°) as in c, except that the maximum number of pulses were now limited to 50 and the isometric peak force was considered at 15° of knee flexion to ensure a continuity in the force-velocity curves.

Our model showed that knee joint angle where the stimulation was initiated affects the shape of the force velocity relationship, and consequently the zero force shortening velocity during shortening velocities (Fig. 10a and 10b). In addition, when the muscle was stimulated throughout the range of motion, our model captured the classical force-velocity behavior during shortening contractions, predicted that the peak forces produced during lengthening contractions are higher than isometric and shortening contractions, and showed that the peak forces initially rise with increases in lengthening velocity and then plateau with further increases (Fig 10c). More quantitatively, the model predicted that the peak force during lengthening contractions was 1.4 times the isomeric peak force (at optimal muscle length) in response to a stimulation frequency of 100 Hz (data were averaged for six subjects tested). This observation was consistent with previously published experimental data that showed that the force during the plateau portion of the eccentric contractions was 1.4 times the force during isometric contractions [45]. Figure 10d shows the effect of limiting the maximum number of pulses to 50. The limitation on the number of pulses being delivered to the muscle affected the shape of the force-velocity curves during shortening and lengthening contractions, which was qualitatively consistent with the experimental data [45,46]. Hence, factors like number of pulses, IPI, and muscle length when the stimulation is initiated, which are important factors in an FES application, affect the shape of the force-velocity curve and should be considered when developing models for a FES application.

During eccentric contractions, the muscle lengthens instead of shortening. Such contractions are characterized by higher forces than isometric and shortening contractions and are important parts of many functional activities, such as walking. Hill-type models that predict eccentric muscle forces use a separate set of equations to describe the behavior of the muscle during eccentric contractions [6]. For the current model only one set of equations were necessary. The reason for the model's ability to predict lengthening contractions was due to the coupling between *G*(*θ*)$\dot{\theta }$ and *C*_*N*_/(*K*_*M *_+ *C*_*N*_) (see Eqn. 13). *G*(*θ*)$\dot{\theta }$ increases with lengthening velocity whereas *C*_*N*_/(*K*_*M *_+ *C*_*N*_) decreases. At a given stimulation frequency, the greater the lengthening velocity the fewer the number of pulses delivered and, hence, smaller the value of *C*_*N*_. The increase in *G*(*θ*)$\dot{\theta }$ with lengthening velocity was, therefore, compensated by a decrease in *C*_*N*_/(*K*_*M *_+ *C*_*N*_) and helped to maintain the peak forces at almost a constant value with increasing velocities in the flat portion of the force-lengthening velocity curve (see Figs. 10c and 10d).

The current model development and verification were done for healthy human quadriceps femoris muscle under isovelocity conditions. Because of the various assumptions made in developing the current model, the model may not be valid when applied to other muscle groups, when muscles become fatigued, or to patient population. For example, the assumptions of having τ_*c *_at a fixed value of 20 ms or that parameter *M *is independent of joint velocity may not be valid for spinal cord injured or stroke patients. In addition, for these and other patient populations reflex activity needs to be considered when predicting the forces in response to electrical stimulation. Also, for the current model to be useful for an FES application like walking, the model needs to predict the angular velocity based on the load and stimulation pattern. Finally, the current modelling work only considered the effect of stimulation frequency and pattern. Future modelling work will also need to predict the effects of stimulation intensity on the forces produced by the muscle.

## Conclusion

This study showed that the current model predicted the forces in response to a wide range of stimulation frequencies and constant velocities in able-bodied human quadriceps muscles. Our model did not assume an *a priori *force-velocity relationship. Rather, the relationship was a natural outcome of modeling the viscoelastic and contractile behavior of the muscle. The range of predictive abilities of the isovelocity model in response to changes in muscle length, velocity, and stimulation frequency for each individual make it ideal for dynamic applications like FES cycling [47]. In FES cycling where an external motor maintains the speed of cycling constant, our model can be used to design stimulation patterns that can produce the targeted level of power output from the muscle.

## Competing interests

The authors declare that they have no competing interests.

## Authors' contributions

RP was involved with mathematical modeling, subject recruitment, data-collection, analysis, and manuscript preparation. SABM and ASW were involved in all aspects of the study, supervised the design and coordination of the study, and provided critical revisions of the manuscript. All authors read and approved of the final manuscript.
